# Possible Predictive Factor of Acute Respiratory Distress Syndrome Development After Mild Traumatic Brain Injury: A Single Rural Trauma Center Preliminary Study

**DOI:** 10.7759/cureus.16508

**Published:** 2021-07-20

**Authors:** Enoch Yeung, Matthew Miller, Cynthia Wung, Robert Behm, Burt Cagir, Paul Granet

**Affiliations:** 1 Surgery, Guthrie Robert Packer Hospital, Sayre, USA; 2 Trauma/Critical Care, Guthrie Robert Packer Hospital, Sayre, USA; 3 Colorectal Surgery, Guthrie Robert Packer Hospital, Sayre, USA; 4 Trauma/Surgical Critical Care, Guthrie Robert Packer Hospital, Sayre, USA

**Keywords:** ards, mild tbi, predictive risk factors, mortality, morbidity

## Abstract

Introduction

Acute respiratory distress syndrome (ARDS) after mild traumatic brain injury (TBI) can be associated with significant morbidity and mortality. This study aimed to evaluate the potential predictive factors of ARDS development following mild TBI in trauma patients.

Methods

A retrospective chart review was done for adult trauma patients with mild TBI (GCS 13-15) requiring admission at our center from 2012 to 2020. Linear regression analysis and chi-square test were utilized to identify independent predictors of the association with ARDS in adults with mild TBI.

Results

A total of 784 mild TBI patients were admitted during the time of interest; 34 patients developed ARDS during their index hospitalization. Patients who had ARDS were more likely to have acute kidney injury (AKI; p < 0.0001), sepsis (p < 0.01), rib fractures (p < 0.05), use of anticoagulants (p < 0.001), deep vein thrombosis (p < 0.001), transfusion during the first 4four hours upon admission (p = 0.01), intravenous fluid (IVF) resuscitation during the first four hours (p <0.05), the first eight hours (p = 0.01), the first 12 hours (p = 0.03), and intubation upon the admission (p < 0.0001). ARDS associated with mild TBI demonstrated a statistically significant increase in mortality during the index hospitalization (p < 0.0001).

Conclusion

ARDS after mild TBI can be associated with significant morbidity and mortality. Key risk factors identified include AKI, sepsis, anticoagulant use, deep vein thrombosis (DVT), transfusion in the first four hours, IVF resuscitation in the first four, eight, and 12 hours, and intubation upon admission.

## Introduction

Traumatic brain injury (TBI) is defined as an alteration in brain function, or other cause of brain pathology, caused by an external force [1]. It is a major cause of disability and mortality among individuals in both developed and developing countries, and it was found to be responsible for 2% of all deaths in the United States. There were about 56,000 TBI-related deaths in the United States in 2014. TBI is the number one cause of death under the age of 40 years and it is a contributing factor to over 30% of all injury-related deaths in the United States [2]. Acute respiratory distress syndrome (ARDS) is a well-described complication of patients admitted for TBI. The Berlin criteria of ARDS include acute onset within seven days, bilateral pulmonary infiltrates consistent with pulmonary edema, and impaired oxygenation not fully explained by cardiac failure and differentiates mild, moderate and severe ARDS based on the PaO_2_ to FiO_2_ ratio. The worldwide incidence of ARDS in severe TBI has been reported to be around 30%, with a 30-40% mortality rate reported in this population [3]. However, the relationship between mild TBI and ARDS is complicated and not completely understood. The development of ARDS after TBI was showed to be associated with a low partial pressure of oxygen in brain tissue [4]. The brain injury leads to systemic release of inflammatory cytokines, which leads to increased permeability and neutrophil invasion into the lungs and subsequent hypoxia due to lung damage along with other factors which further exacerbate the brain injury [5]. ARDS following TBI in trauma patients drives up healthcare costs, prolongs ICU stays, and is associated with more overall complications [6].

Despite the correlation between TBI and ARDS being explored in numerous studies, the factors that drive this association are still unknown [7]. Most of the published literature examines risk factors for ARDS development after severe TBI, in which patients were usually intubated for airway protection [8]. However, there are few studies on the relationship between mild TBI and ARDS development. Mild traumatic brain injury is defined as brain injuries in which the Glasgow Coma Scale (GCS) score 30 minutes after the traumatic injury is 13 to 15, loss of consciousness of equal to or less than 30 minutes, and the duration of post-traumatic amnesia is no less than or equal to 24 hours [9]. Currently, no validated tools exist to predict which mild TBI patients are at the highest risk for ARDS. Given the immense morbidity and mortality associated with ARDS and the tremendous increase in healthcare cost, an accurate predictive protocol for the likelihood of ARDS occurring after mild TBI would be extremely useful for critical care management.

This study explores the possible predictive risk factors that are significantly associated with ARDS development in patients who presented to our center following mild TBI. We aimed to add our study to a body of literature that will determine how clinicians can use these associations to better triage and treat patients with mild TBI and improve prevention and treatment of subsequent ARDS development.

## Materials and methods

Data collection

This study was a retrospective clinical study conducted with patient chart review in a single rural trauma center following approval by the Institutional Review Board, Guthrie Robert Packer Hospital. Eligible patients were captured using an administrative surgical registry. The IRB approved the waiver of consent. Comprehensive demographic and clinical data were collected including patient age, gender, baseline comorbidity, length of stay in hospital, length of stay in ICU, duration of intubation, and trauma-related complications. We identified 784 consecutive patients who enrolled in a retrospective cohort study at our institution, a rural level II trauma center between July 2012 and March 2020 in a single trauma center. All cases were cross-referenced with CPT codes to only include those associated with mild TBI. Patients admitted for head injury with admission GCS 13-15 were included. An adjudication protocol for diagnosis of ARDS was carried by two trauma surgeons to identify cases according to the Berlin definition in the first seven days of admission as previous description [10]. Fluid resuscitation was defined as intravenous fluid given at 1 liter or more bolus of normal saline or lactated Ringer solution [11]. Blood transfusion was defined as transfusion of blood products including red blood cells, fresh frozen plasma, platelets, or cryoprecipitate.

Statistical analysis

In this study, continuous data are represented by the mean with standard deviation from the mean in a Gaussian normal distribution. The categorical variables were compared as incidence rate and presented as percentage. In all analyses, α < 0.05 was considered significant. Univariate logistic regression models were used to test the association between all biologically plausible predictors and ARDS in mild TBI patients. Univariate comparisons were made using Student’s t-test and chi-square test for continuous variables and categorical variables, α < 0.05 was considered significant. A univariate logistic regression model is used to test for differential risk of ARDS by patient characteristics. Mortality was defined as the last follow-up with evidence of death from the electronic record. It was evaluated with chi-square test, α < 0.05 was considered significant. Statistical analysis was performed using the Prism version 8 software (La Jolla, CA: GraphPad Software).

## Results

Patient demographics and clinical characteristics

A total of 784 patients were defined as having a mild TBI on admission and therefore met inclusion criteria. Patient characteristics and biochemical parameters are summarized in Table 1. The median age of the patients in the non-ARDS and ARDS groups were 69 and 74 years old (mean age +/- SD: non-ARDS: 63.6 +/- 21.88, ARDS: 72.38 +/- 14.76), respectively. There were 448 (60%) and 23 (68%) males in non-ARDS and ARDS groups, respectively. There were 400 (53%) and 22 (65%) smokers in non-ARDS group and the ARDS group, respectively. The mean BMI of the non-ARDS and ARDS groups were 26.44 kg/m^2^ and 29 kg/m^2^, respectively. The mean white cell count upon admission of non-ARDS and ARDS groups were 27.3 k/uL and 28.5 k/uL, respectively (reference range: 4.23-9.07 k/uL). The mean hematocrit upon admission of non-ARDS and ARDS groups were 37.37% and 31.6%, respectively (reference range: 40.1-51%). The creatinine/blood urea nitrogen (BUN) upon admission of non-ARDS and ARDS group were 0.9/17.6 and 1.2/38.6 (mg/dL mg/dL^-1^), respectively (reference range: creatinine: 0.8-1.5mg/dL, BUN: 9-20mg/dL). The associated injuries and pre-existing cardiovascular conditions of both cohorts are listed in Table 2. The diagnosis of the involved head injuries is summarized in Table 3. The admission GCS score is summarized in Table 4. The percentage of positive admission CT head findings is summarized in Table 5.

**Table 1:** Patient characteristic and biochemical parameters upon admission ARDS: acute respiratory distress syndrome; WBC: white blood cell; HCT: hematocrit; Cr: creatinine; BUN: blood urea nitrogen

Patient characteristic					
Patients	Non-ARDS		ARDS		P-value
N	N		N		
784	750		34		
Gender					
Male (%)	448 (60%)		23 (68%)		
Female (%)	302 (40%)		11 (32%)		
Smoker (%)	400 (53%)		22 (65%)		0.221
Drinker (%)	259 (35%)		9 (26%)		0.363
Biochemical parameters					
	Mean +/- SD	Median, IQR	Mean +/- SD	Median, IQR	
Age	63.6 +/- 21.88	69, (50-81)	72.38 +/- 14.76	74, (25-83.75)	0.057
BMI (kg/m^2^)	27.3 +/- 6.2	26.44, (23.3-30.2)	28.5 +/- 7.4	29, (22.8-33.2)	0.258
WBC (k/uL)	9.2 +/- 3.5	8.7, (6.7-10.8)	10.4 +/- 3.8	10, (7.5-12.9)	0.080
HCT (%)	37.4 +/- 5.6	37.9, (33.5-41.1)	31.6 +/- 6.3	29, (26.6-35.6)	0.273
Cr (mg/dL)	0.9 +/- 0.7	0.8, (0.7-1)	1.3 +/- 1.3	1, (0.6- 1.1)	0.056
BUN (mg/dL)	17.6 +/- 10.4	15, (12-15)	38.6 +/- 42.5	24, (15-43)	0.049

**Table 2:** Associated injury and pre-existed cardiovascular condition ARDS: acute respiratory distress syndrome; CHF: congestive heart failure; AFIB: atrial fibrillation The diagnoses of cardiovascular disease are not added up to 100% because only some patients in the cohort have those conditions.

Associated Injury			
	Non-ARDS	ARDS	P-value
N	750	34	
Humerus fracture	7 (0.9%)	0	
Femur fracture	3 (0.4%)	0	
Blunt cardiac injury	3 (0.4%)	0	
Spinal fracture	18 (2.4%)	3 (8.8%)	0.058
Pelvic fracture	0	1 (2.9%)	
Perforated bowel	0	0	
Laceration	179 (24%)	6 (17.6%)	0.054
Pre-existed cardiovascular condition			
CHF	127 (17%)	7 (20.6%)	0.58
AFIB	155 (21%)	11 (32.4%)	0.1
Stroke (pre-exiting)	4 (0.5%)	2 (5.9%)	<0.05
Mech valve	110 (15%)	6 (17.6%)	0.62

**Table 3:** Diagnosis of head injury ARDS: acute respiratory distress syndrome The diagnoses of head injury are not added up as 100% because some patients have more than one diagnosis.

Diagnosis of Head Injury		
	Non-ARDS	ARDS
N	750	34
Loss of consciousness	703 (94%)	30 (88.2%)
Subarachnoid hemorrhage	215	5
Epidural hemorrhage	8	0
Subdural hemorrhage	253	19
Contusion/concussion	69	1
Skull fracture	67	2
Skull laceration	26	2
Diffuse traumatic brain injury	9	1
Unspecific head injury	129	4

**Table 4:** Admission GCS score GCS score: Glasgow Coma Scale score; ARDS: acute respiratory distress syndrome

Admission GCS Score		
	Non-ARDS	ARDS
N	750 (%)	34(%)
GCS 13	28 (3.7%)	4 (11.8%)
GCS 14	120 (16%)	9 (26%)
GCS 15	602 (80.3%)	21(62%)

**Table 5:** Positive admission CT head percentage ARDS: acute respiratory distress syndrome

Positive Admission CT Head Percentage			
	Non-ARDS	ARDS	P-value
N	750 (%)	34 (%)	
Positive CT head %	255 (34%)	19 (56%)	0.015

Predictive risk factors of developing ARDS

**Figure 1:**
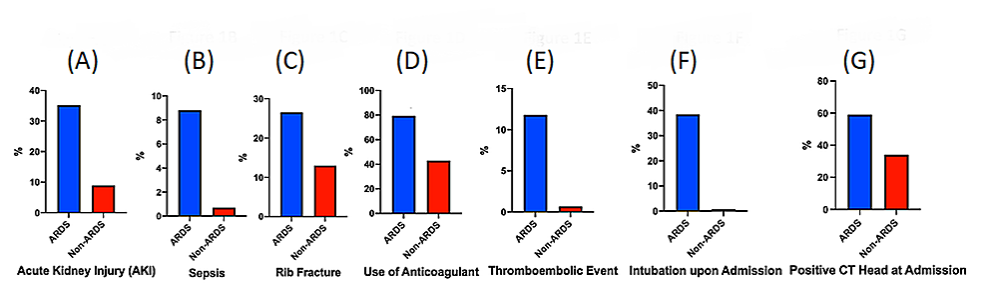
Predictive risk factors of developing ARDS in trauma patient with mild TBI ARDS: acute respiratory distress syndrome; TBI: traumatic brain injury

There were several possible risk factors for ARDS development identified. We noted that the incidence of acute kidney injury (AKI) was significantly higher than that of non-ARDS (ARDS: 35.2% vs non-ARDS: 8.9%, OR: 5.6, CI: 2.651-11.81, p < 0.0001) (Figure 1A). The incidence of sepsis was significantly higher than that of non-ARDS (ARDS: 8.8% vs non-ARDS: 0.7%, OR: 14.4, CI: 3.647-57.76, p = 0.003) (Figure 1B). The incidence of rib fracture in the ARDS group was significantly higher than that of non-ARDS (ARDS: 26.5% vs non-ARDS: 13.0%, OR = 2.4, CI: 1.066-5.255, p =0.03) (Figure 1C). The use of anticoagulation prior to the injury in the ARDS group was significantly higher than that of non-ARDS (ARDS: 79.4% vs 43.0%, OR: 5.1, CI: 2.216-12.33, p < 0.001) (Figure 1D). Also, we observed that the incidence of thromboembolic events in the ARDS group was significantly higher than that of non-ARDS (ARDS: 11.8% vs 0.67%, OR:19.9, CI: 5.782-70.37, p = 0.0003) (Figure 1E). The incidence of intubation upon admission in the ARDS group was found to be significantly higher than that of non-ARDS (ARDS: 38.4% vs 0.8%, OR: 76.8, CI: 26.23-222.4, p < 0.0001) (Figure 1F). The incidence of positive CT head scan admission was significantly higher than that of non-ARDS (ARDS: 55.9%% vs 34.0%, OR=2.46, CI: 26.23- 222.4, p =0.015) (Figure 1G).

Fluid resuscitation and transfusion of blood products by ARDS status

**Figure 2:**
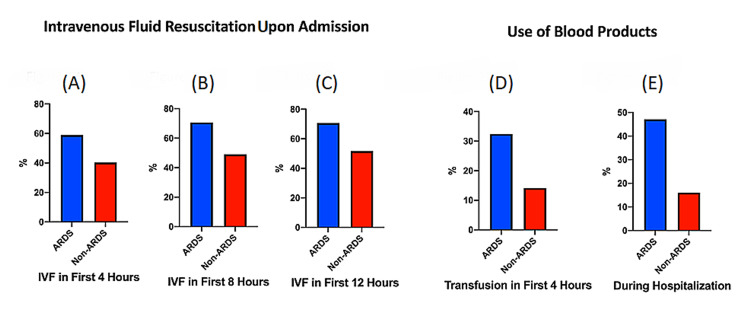
Fluid resuscitation and transfusion of blood products by ARDS status ARDS: acute respiratory distress syndrome; IVF: intravenous fluid

In this study, we noted the intravenous fluid resuscitation and transfusion of blood products were the possible risk factor of ARDS in mild TBI trauma patient. The intravenous fluid resuscitation and blood transfusion were defined in the Materials & Methods section. The patients who had intravenous fluid resuscitation in the first four hours from admission had a higher risk of developing ARDS (ARDS: 58.8% vs 40.3%, OR=2.1, CI: 1.068-4.274, p < 0.05) (Figure 2A). The patient who had intravenous fluid resuscitation in the first eight hours from admission had a higher risk of developing ARDS (ARDS: 70.6% vs 49%, OR=2.5, CI: 1.224-5.170, p = 0.01) (Figure 2B). The patient who had intravenous fluid resuscitation in the first 12 hours from admission had a higher risk of developing ARDS (ARDS: 70.6% vs 51.6%, OR =2.3, CI: 1.088-4.599, p = 0.03) (Figure 2C). Regarding the blood product transfusion, the patient who had blood transfusion in the first four hours upon admission had a higher risk of developing ARDS (ARDS: 32.4% vs 14.1%, OR=2.9, CI: 1.339-6.069, p = 0.01) (Figure 2D). The patient who had blood transfusion during the hospitalization had a higher risk of developing ARDS (ARDS: 47.1% vs non-ARDS: 16%, OR =4.5, CI: 2.290-9.179, p < 0.0001) (Figure 2E).

Clinical outcomes and clinical predictive factor by ARDS status

Mortality and Length of Stay

**Figure 3:**
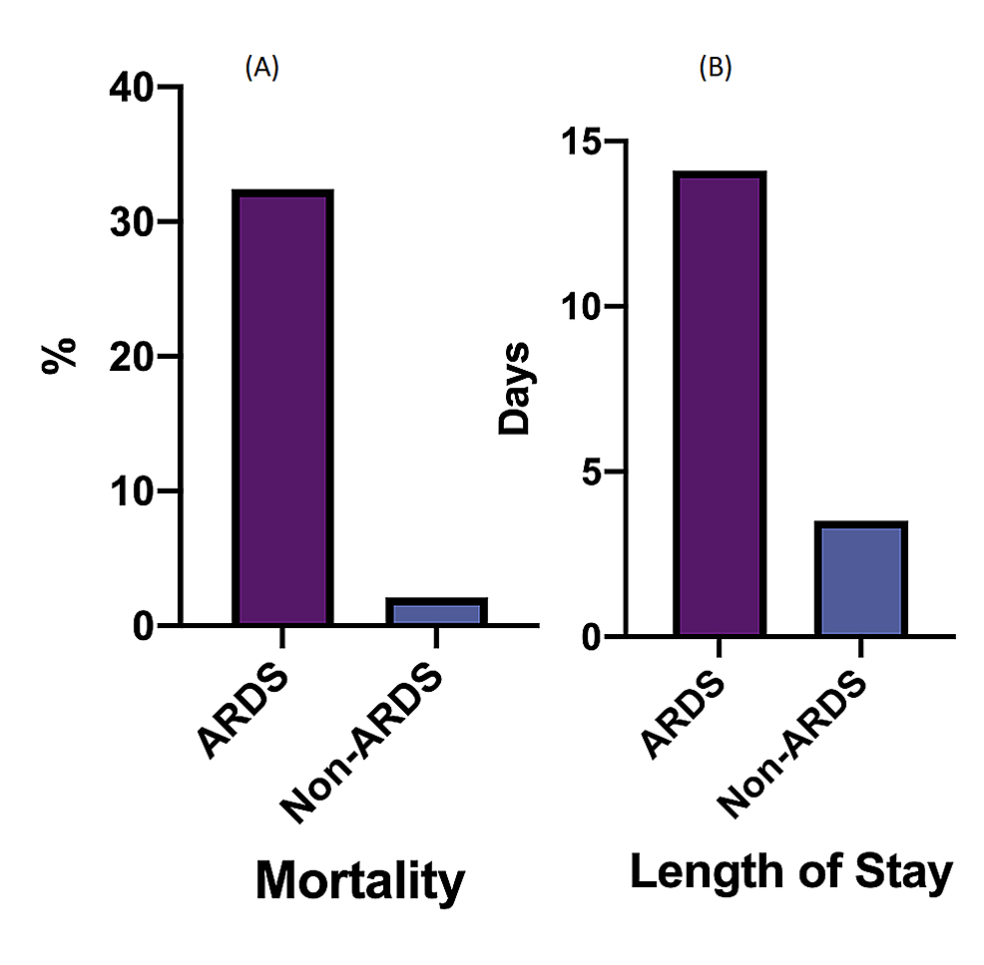
Clinical outcomes by ARDS status ARDS: acute respiratory distress syndrome

Although this study was not meant to evaluate the clinical outcomes. We noted that the mortality in the ARDS group was significantly higher than that of non-ARDS (ARDS: 32.4% vs non-ARDS: 2.13%, OR: 21.9, CI: 9.325 - 52.92 p < 0.0001) (Figure 3A). The mean length of stay (days) was significantly longer than that of non-ARDS (ARDS: 14.1 days vs non-ARDS: 3.5 days, p < 0.0001) (Figure 3B).

Subgroup analysis for rib fracture cohort

**Figure 4:**
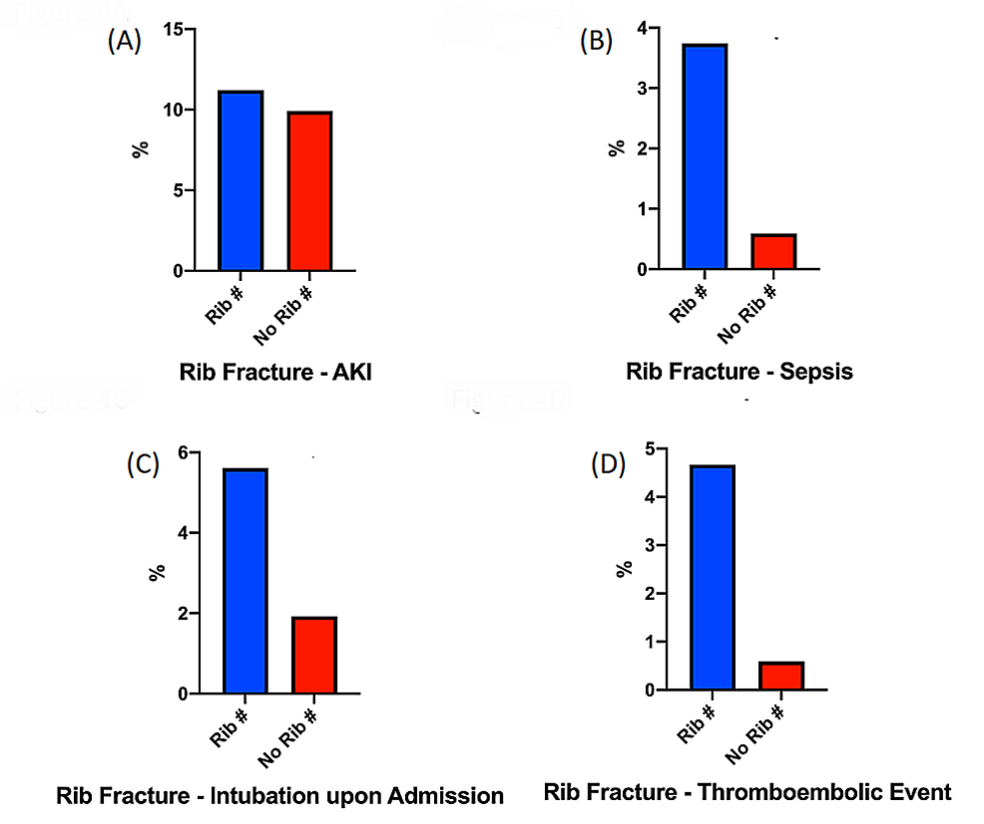
Subgroup analysis by rib fracture status AKI: acute kidney injury

Since rib fracture has been known to be an independent risk factor of ARDS development. We performed subgroup analysis for the cohort according to the rib fracture status. There was no difference noted in the incidence of AKI between rib fracture and the non-rib fracture group (rib fracture: 11.2% vs non-rib fracture: 9.9%, p > 0.05) (Figure 4A). There was higher incidence of sepsis in the rib fracture group (rib fracture: 3.7% vs non-rib fracture: 0.6%, OR: 6.5, CI: 1.9-22.7, p < 0.01) (Figure 4B). There was higher incidence of intubation in the rib fracture group (rib fracture: 5.6% vs non-rib fracture: 1.9%, OR: 3.0, CI: 1.2- 7.6, p < 0.05) (Figure 4C). There was higher incidence of thromboembolic events in the rib fracture group (rib fracture: 4.7% vs non-rib fracture: 0.6%, OR: 8.3, CI: 2.4- 27.1, p < 0.05) (Figure 4D).

Non-Predictive Parameters

There was no difference noted in the incidence of ICU admission, the incidence of hypotension upon admission (defined as systolic blood pressure <90 mmHg), the incidence of pre-existing chronic obstructive pulmonary disease (COPD), the incidence of smoking, and the incidence of drinking between ARDS and the non-ARDS groups (p > 0.05).

## Discussion

ARDS after mild TBI is associated with significant morbidity and mortality. Given that mild TBI is one of the most common indications for trauma admission, having a clinical predictive tool for which patients will likely develop ARDS would be very clinically valuable. The correlations we found between these two common pathologies in our trauma population provide interesting data that can be further investigated by future studies. In our level II trauma center, 34 of 784 mild TBI patients during their initial hospitalization were identified. Within this group, several clinical parameters were noted to be more prevalent in those patients who developed ARDS. Among these associations were acute kidney injury, sepsis, rib fractures, use of anticoagulants, deep vein thrombosis, transfusion during the first four hours of admission, intravenous fluid (IVF) resuscitation during the first four, eight, and twelve hours of admission, and intubation on admission.

We noted that patients with mild TBI who were intubated during initial hospitalization had much higher rates of developing ARDS than those who were not initially intubated (38.4% vs. 0.8%, p < 0.0001). Kangelaris et al. found that patients with early intubation in ARDS were associated with higher rates of organ dysfunction and shock though late intubation was reported to be associated with higher mortality [12]. Our study results are consistent with this study finding in that the intubated cohort had a complicated clinical course including organ failure and sepsis. Although the causative relationship between early intubation and organ failure is still being investigated, our results may be a useful reference for further study of mechanical ventilation association with organ failure.

We noted that patients with rib fracture were more likely to have ARDS than those who did not (26.5% vs 13.0%, p = 0.03). Tignanelli et al. recently reported that having a rib fracture is an independent risk factor of ARDS development [13]. The group also reported individual number of rib fractures from one rib fracture to more than eight rib fractures also increased risk of ARDS development (p > 0.001). In our study, a subgroup analysis regarding rib fractures showed there was a higher incidence of sepsis and intubation in the rib fracture group. There is a possibility that the presence of rib fractures can be a confounding factor for the development of sepsis as a sequela of pulmonary complication from rib fractures. De Freitas Cardoso et al. showed the executive dysfunction in mild TBI was repaired (visual memory test learning component, p < 0.001) [14]. The capability of using incentive spirometry, which is an important treatment for rib fractures, can be less efficient in mild TBI patients. The combined effect of reduced learning ability from mild TBI may increase the risk of pneumonia and sepsis. Further investigation regarding the potential correlation between rib fractures and ARDS in patients with mild TBI is needed to better understand any causative relationship.

The present study showed AKI was significantly more prevalent in those mild TBI patients who developed ARDS when compared to those who did not develop ARDS (35.2% vs 8.9%, p < 0.0001). Li et al. demonstrated that the kidneys and lungs are intimately linked and damage of one of these organs often adversely affects the other [15]. One meta-analysis by van den Akker et al. showed there is a linkage between mechanical ventilation with AKI risk among critically ill patients [16]. With the reduced preload due to increased thoracic pressure, the resultant hemodynamic effects may lower the kidney’s perfusion hence the glomerular filtration rate and clearance of free water [17]. Annat et al. demonstrated the hormonal changes in the renin-angiotensin system and sympathetic nervous output observed during positive pressure ventilation leading to lower GFR, sodium excretion, and urine output [18]. Rocha et al. showed that respiratory failure and AKI are intimately linked, though the group does not establish a clear picture of how the timing of initial mechanical ventilation relates to the onset of AKI [19]. The biotrauma factors from mechanical ventilation may affect much more than the lungs; this release of inflammatory cytokines can lead to systemic inflammation and organ dysfunction [20]. Our study found that mild TBI patients diagnosed with sepsis were drastically more likely to develop ARDS than those patients who did not exhibit sepsis (8.8% vs. 0.7%, p = 0.003). Iriyama et al. showed soft tissue infection and ICU admission were the possible risk modifier of ARDS in patient with sepsis [21]. In our study, ICU admission was not noted to be a significant risk factor of sepsis. However, there were about 30% of ARDS patients had soft tissue infection in ARDS in our cohort. We believe this finding is consistent with the previous study. In our subgroup analysis, there was a significant portion of patients with rib fractures who developed ARDS. There is a possibility that the rib fractures may play a role in the sepsis process such as pulmonary contusion or prolonged mechanical ventilation [22]. Other studies have shown that refractory sepsis and multiorgan failure in ARDS are actually much more likely to predict a worse outcome [23]. Our results of increased mortality in the ARDS group may also be consistent with these studies. However, the component of mild TBI in those patients warrants further study to clarify. 

We found that there was a significant increase in the development of ARDS based on whether our mild TBI patients received fluids at the four, eight, or 12-hour marks, respectively (58.8% vs. 40.3%, p < 0.05; 70.6% vs. 49%, p = 0.01; 70.6% vs. 51.6%, p = 0.03). Hendrickson et al. demonstrated that in the era of modern hemostatic resuscitation practices, the following variables are associated with a higher risk of developing ARDS: male sex, increased severity of the head injury, and early administration of crystalloids and platelets after severe isolated TBI [24]. The association between crystalloids and ARDS is not unique to the cohort of isolated TBI. Howard et al. reported this association among all intubated trauma patients [25]. One of the possible explanations is the deleterious effects of aggressive resuscitation on acid-base status, metabolism, and oxygen exchange [26]. Although not much is yet published on the association between ARDS and early fluid resuscitation in patients with mild TBI, this study's results showing adverse effects of early intravenous fluids are consistent with the previous study. 

The present study noted that there is a significant increase in the development of ARDS in mild TBI patients who received blood transfusion within four hours of admission compared to those who did not (32.4% vs. 14.1%, p = 0.01, respectively). Many studies focus on platelets when looking at adverse pulmonary outcomes related to transfusion. Larsen et al. demonstrated the platelet transfusion has untoward effects on the systemic response to injury such as ARDS [27]. Watson et al. showed that FFP was independently associated with the development of MOF and ARDS whereas platelets were not in patients with blunt injury and hemorrhagic shock [28]. However, a multicenter study by Spinella et al. showed that high platelet ratio transfusion improved survival in trauma patients with TBI patients whereas high platelet ratio transfusion improved survival in trauma patients without TBI [29]. In our study, we did not have a large enough sample size to distinguish the effects of different blood products. Platelets are most often transfused along with other blood products; the present study which showed a correlation between transfusion and ARDS may inaccurately imply that all blood products increased risk for ARDS, while platelets may be the primary culprit. The present study showed that patients who were on anticoagulants prior to trauma had higher rates of ARDS. Interestingly, the incidence of thromboembolic events was also higher in the ARDS group. These findings seem to highlight the importance of baseline cardiovascular function on clinical outcomes in patients who experience TBI. However, the differences in cardiovascular risk did not reach statistical significance (Table 2). This lack of significance is likely due to our relatively small cohort sizes. Further studies with higher power are warranted.

Though it is not the primary outcome, we noted that ARDS associated with mild TBI demonstrated a statistically significant increase in mortality during the index hospitalization (32.4% vs 2.13%, p < 0.0001). Prior studies using the Berlin criteria have focused on severe TBI. In severe TBI, ARDS has been shown to be significantly associated with increased mortality by the study from Aisiku et al. [3]. ARDS has already been shown to be a predictor of mortality in other conditions such as burn and severe TBI. The present study has shown this positive association even in mild TBI. This possible correlation can lead to more prompt recognition of ARDS and initiation of lung-protective strategies earlier to optimize critical care management.

The limitations of this study include that it is a single-center study. The cohort population also lacks generalizability. The population of this cohort may not be able to represent the general population. The validity of the study needs to be studied by multi-center study. In this study, the positive CT finding in the ARDS group was about 56%, which was higher than that reported in systemic analysis by Marincowitz et al. [30]. Another limitation is the retrospective nature of this study. There are several confounding factors such as the baseline comorbidities that were not controlled for in this study. Those comorbidities can have a tremendous effect on the outcomes including mortality. Associated injuries of each cohort are important considerations when evaluating the development of further pathologies (Table 2). In this study, the number of patients with associated injuries was not large enough for subgroup analysis. Further investigation of the association between various injuries and ARDS development is needed. Additionally, long-term follow-up of neurological function would better elucidate the relationship between mild TBI and ARDS. Unfortunately, most of our patients had three or fewer follow-up appointments with our trauma team. However, we will improve upon this issue by utilizing virtual appointments in the future. Another limitation of our study is the absence of a trauma score severity. This score would add valuable insight to the overall clinical picture of our patients. Unfortunately, the severity of individual injuries was not included in a portion of our patient population. In order to have an accurate and consistent report, we were unable to analyze this parameter. The present study serves as a preliminary study, and we will collect Injury Severity Scores (ISSs) in all our trauma patients going forward.

One of the limitations of this study is that the number of patients restricted the possibility of subgroup analysis based on different patient clinical parameters and the further classification of the severity of ARDS according to the Berlin classification. Subgroup analysis and possible propensity score analysis of different parameters with the severity of the ARDS will be helpful to delineate the correlation between those factors and the degree of physiological insult from ARDS. Further large cohort study with collaboration with other institutions is suggested for further subgroup analysis for a clear picture of how the clinical parameters may influence the clinical outcome in mild TBI of development of ARDS. Another way to improve upon this study would be to apply a propensity matching analysis to the relationship between mild TBI and ARDS. In future work, we will include this analysis in order to add to the significance.

Though we included rib fractures as one of the clinical parameters being investigated, there is other clinically important pulmonary-related trauma complex that can increase the risk of ARDS. Another limitation of this study is the lack of a causative relationship between rib fracture and sepsis. As sepsis is known to be a risk factor of ARDS, a subgroup analysis with a larger trauma patient population is suggested to investigate the correlation. The compliance of the use of incentive spirometry and the number of rib fractures were also helpful parameter to further clarify the possible role of rib fracture in this complex pathology (ARDS in mild TBI). We also feel that the intravenous fluid resuscitation risk stratification has room for improvement. The amount of fluid and the individual bodyweight of the patient may play an important role to find out if the patient has a risk of over-resuscitation. Those parameters are suggested to be included in our next follow-up study. Furthermore, the type and the volume of blood products can be categorized to avoid confusion from the transfusion-related lung injury as we know the platelet is known to cause transfusion-related acute lung injury (TRALI). All those categorizations in a larger cohort will be useful for further study. Lastly, this study does not prove causal relationships between any of these factors and the development of ARDS. We would suggest a further study to examine the causation between those risk factors to guide further clinical management.

Despite the limitations, this study aimed to provide a preliminary study result regarding patients who presented with mild TBI, which is accurately diagnosed based on Berlin classification since July 2012 for a homogenous cohort. We hope to take the initiative to look at those clinical parameters that were potentially significantly correlated with the development of ARDS in mild TBI patients. These parameters may contribute to the creation of a predictive tool to help clinicians better prevent and treat mild TBI patients who are at risk for developing ARDS.

## Conclusions

Mild TBI is a very common diagnosis in trauma patient while ARDS is a very morbid complication; therefore, any factors that may predict and help prevent this complication warrant exploration. In this study, we presented the first study in the literature that examines the development of ARDS in the mild TBI patient population specifically. We described the important epidemiologic findings as a preliminary study that may guide translational research to prevent and treat ARDS after mild TBI, and thereby improve clinical outcomes in this vulnerable population. Future studies with larger sample sizes and multiple institutions are warranted to further decipher the utility of knowing these relationships.
